# Consequences of the COVID-19 pandemic on lung cancer care and patient health in a German lung cancer center: results from a cross-sectional questionnaire

**DOI:** 10.1186/s12931-022-01931-z

**Published:** 2022-01-29

**Authors:** Julia Walter, Laura Sellmer, Kathrin Kahnert, Rosemarie Kiefl, Zulfiya Syunyaeva, Diego Kauffmann-Guerrero, Farkhad Manapov, Christian Schneider, Juergen Behr, Amanda Tufman

**Affiliations:** 1 Department of Internal Medicine V, Thoracic Oncology Centre Munich, Ludwig-Maximilians University Munich (LMU), Ziemssenstraße 1, 80336 Munich, Germany; 2 German Center for Lung Research (DZL CPC-M), Aulweg 130, 35392 Gießen, Germany; 3 Department of Radiation Oncology, Thoracic Oncology Centre Munich, Ludwig-Maximilians University, Marchioninistraße 15, 81377 MunichMunich, Germany; 4 Department of Thoracic Surgery, Thoracic Oncology Centre Munich, Ludwig-Maximilians University Munich, Marchioninistraße 15, 81377 Munich, Germany

**Keywords:** Corona virus, SARS-CoV-2, Thoracic malignancies, Social distancing, Quarantine

## Abstract

**Background:**

The novel coronavirus SARS-CoV-2 has caused a global COVID-19 pandemic, leading to worldwide changes in public health measures. In addition to changes in the public sector (lockdowns, contact restrictions), hospitals modified care to minimize risk of infection and to mobilize resources for COVID-19 patients. Our study aimed to assess the impact of these measures on access to care and behaviour of patients with thoracic malignancies.

**Methods:**

Thoracic oncology patients were surveyed in October 2020 using paper-based questionnaires to assess access to ambulatory care services and tumor-directed therapy during the COVID-19 pandemic. Additionally, behaviour regarding social distancing and wearing of face masks were assessed, as well as COVID-19 exposure, testing and vaccination. Results are presented as absolute and relative frequencies for categorical variables and means with standard deviation for numerical variables. We used t-test, and ANOVA to compare differences in metric variables and Chi^2^-test to compare proportions between groups.

**Results:**

93 of 245 (38%) patients surveyed completed the questionnaire. Respiration therapy and physical therapy were unavailable for 57% to 70% of patients during March/April. Appointments for tumor-directed therapy, tumor imaging, and follow-up care were postponed or cancelled for 18.9%, 13.6%, and 14.8% of patients, respectively. Patients reported their general health as mostly unaffected. The majority of patients surveyed did not report reducing their contacts with family. The majority reduced contact with friends. Most patients wore community masks, although a significant proportion reported respiratory difficulties during prolonged mask-wearing. 74 patients (80%) reported willingness to be vaccinated against SARS-CoV-2.

**Conclusions:**

This survey provides insights into the patient experience during the second wave of the COVID-19 pandemic in Munich, Germany. Most patients reported no negative changes to cancer treatments or general health; however, allied health services were greatly impacted. Patients reported gaps in social distancing, but were prepared to wear community masks. The willingness to get vaccinated against SARS-CoV-2 was high. This information is not only of high relevance to policy makers, but also to health care providers.

**Supplementary Information:**

The online version contains supplementary material available at 10.1186/s12931-022-01931-z.

## Background

The novel coronavirus SARS-CoV-2 has caused a global COVID-19 pandemic, leading to worldwide changes in public health measures. In addition to changes in the public sector (lockdowns, contact restrictions), hospitals modified care to minimize risk of infection and to mobilize resources for COVID-19 patients. Patients with thoracic malignancies may be particularly affected by public health measures. On the one hand these patients may be at risk of severe COVID complications due to advanced age and comorbidities [1, 2]. On the other hand, patients with thoracic tumors, particularly with locally advanced or metastatic disease, are in need of regular therapy and follow-up care including tumor imaging. The European Society for Medical Oncology (ESMO) was quick to implement new guidelines to help caretakers adjust and prioritize outpatient and inpatient visits for therapy, imaging and follow-up care for cancer patients [3]. They allocated high priority for visits for the continuation of in- and outpatient therapies like intravenous chemotherapy and immune therapy as well as radiotherapy, and imaging for patients at a high risk for relapse and to confirm a cancer diagnosis [3]. Follow-up visits for patients with a low or intermediate risk for relapse after radical therapy were allocated a lower priority [3]. Where possible, teleconsulting was recommended. These recommendations resulted in a conversion of in person visits to phone or video consultations in 52% of patients with contacts to a hospital in March and April 2020 according to a study from The Netherlands [4]. Other studies report substantial decreases in cancer screening and diagnosis as well as treatment [5, 6]. In a global survey of oncology care centers, 36.5% of the respondents reported exposure to harm for patients due to interruption of cancer-specific care and 39.0% reported harms due to interruption of non-cancer related care. 46.3% of centers indicated that more than 10% of their patients missed at least one cycle of chemotherapy [7]. So far, there are no studies measuring gaps in care due to the pandemic in Germany, specifically Bavaria, which was one of the most affected regions during the first wave. Therefore, the aim of our study was to (1) assess the amount of disruption in ambulatory and cancer-related care, (2) assess subjective effects on patients’ health; and (3) assess patient compliance with social distancing guidelines and community masks as well as willingness to be vaccinated in patients with thoracic malignancies.

## Methods

### Study design, patient cohort and data collection

In this cross-sectional study we assessed the experience of thoracic oncology patients during the COVID-19 pandemic concerning access to healthcare, effects on their health, and COVID-19 exposure.

We considered all patients whose cases had been reviewed in the multidisciplinary thoracic oncology tumor board of the thoracic oncology center Munich during the last 3 years, excluding those who had been surveyed as part of our longitudinal telephone-based COVID-19 survey [8]. In addition, we excluded patients treated primarily at other centers (e.g. patients who consulted us for second opinions, patients presented to the tumor board by external physicians, and patients with a documented change of health care provider), patients with metastatic disease who had not had contact with our center during the previous 6 months, and lastly patients who were presented to the board with pulmonary diseases other than lung cancer.

We sent out paper-based questionnaires, patient information, and consent forms to the identified patients. Patients had one month to complete the questionnaire. The questionnaire and the signed consent form were then sent back in a pre-paid envelope. Additional medical history details (histology, stage, current state of disease, current therapy, and comorbidities) were obtained from electronic patient records. Charlson comorbidity score was calculated from ICD codes found in the electronic patient records [9–11]. The primary thoracic oncology diagnosis was not included as a comorbidity for calculation of the Charlson comorbitity index(CCI).

### Ethics

Approval for this cross-sectional non-interventional study was obtained from the Ethics Committee of the Ludwig-Maximilians University. The study was conducted in accordance with the Declaration of Helsinki, Good Clinical Practice guidelines, and local ethical and legal requirements.

### Questionnaire

The questionnaire was designed by our study team (including an epidemiologist, a biologist, and a thoracic oncology specialist). It was designed to evaluate the impact of the COVID-19 pandemic on access to ambulatory care, out- and inpatient tumor-directed care, behavior and effects on subjective general health during the COVID-19 pandemic, and questions directly related to COVID-19. In the first block of questions we asked about availability of physical therapy, respiration therapy, self-help groups, and medical sports courses during the height of the pandemic (March/April 2020). In the second block we asked about appointments for tumor imaging, out- and inpatient intravenous therapy (chemotherapy or immunotherapy), radiotherapy, tumor surgeries, and follow-up visits during oral therapy (tyrosine kinase inhibitors) and after other tumor-directed therapies, previously scheduled for March and April 2020. The third block of the questionnaire consisted of questions regarding social distancing behavior in private life (meeting family and friends) and relating to doctor visits (visits to primary care physicians (GP), and pneumologists/oncologist or other specialists), effects of the pandemic on patients’ subjective general health and mask wearing habits and difficulties in mask wearing. These questions were asked on a 5 level Likert scale ranging from strong agreement to strong disagreement. The fourth question block directly related to COVID-19 exposure, polymerase chain reaction (PCR) and antibody testing, as well as the willingness to get vaccinated. Patient demographics included age, gender, education, working status, and household size For validation purposes, the questionnaire was reviewed by a thoracic oncology physician as well as an external public health expert, and tested on several internal and external test subjects.

### Statistical analysis

All data were pseudonymized prior to analysis. We reported descriptive statistics as absolute and relative frequencies for categorical and ordinal variables and as mean with standard deviation for all metric variables. Metric variables were tested for normal distribution using Shapiro–Wilk-Test. As the chance of the Shapiro–Wilk-Test to be statistically significant with increasing n [12], we additionally performed graphical inspection of the histogram and the QQ-Plot. Respondents and non-respondents were compared using, Students t-test for age and Chi^2^-test, for gender, histology and stage. We performed tests of association with the degree of agreement and age, CCI, gender, metastases status, and education level for the ten questions of question block number three (social distancing and behavior). We used analysis of variance (ANOVA) test for age and CCI, and Chi^2^-test for gender, metastases status, and education level. We applied a threshold of α < 0.05 for significance in all analyses.

Data analysis was performed using R Version 4.0.0. Tables and figures were created in Microsoft Excel.

## Results

### Patient population and demographics

**Fig. 1:**
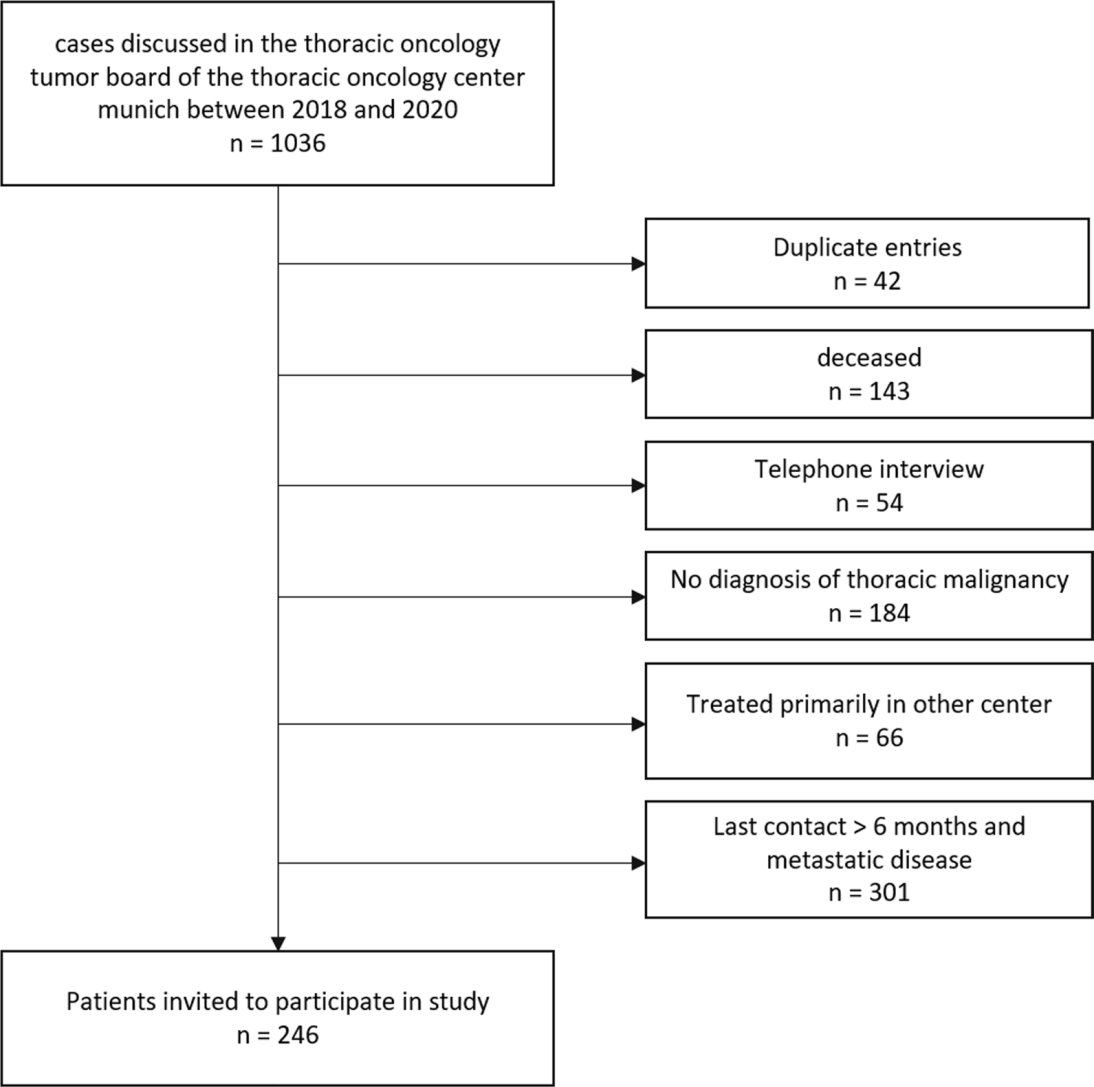
Patient flow diagram. Display of patient flow diagram, and reasons for excluding patients. The basic population consisted of all patients reviewed by the multidisciplinary thoracic oncology tumor board between 2018 and 2020, excluding patients with duplicate entries, deceased patients, patients included in longitudinal telephone-based COVID-19 survey, patients treated primarily at other centers, patients with metastatic disease who had not had contact with our center during the previous 6 months, and patients without thoracic malignancies

Of the 1036 cases discussed in the tumor board of our center during the past 3 years, 790 were excluded due to the exclusion criteria for this study. The patient flow diagram in Fig. 1 shows the reasons for exclusion of patients. Of the remaining 246 patients receiving the questionnaire, 93 (38%) completed the questionnaire and gave consent to be included in this study.

Mean patient age was 66.7 years and 60.2% of patients were male. Information on level of education, working status, histology, stage at diagnosis, current disease status and therapy, as well as comorbidity are displayed in Table 1. Characteristics of respondents were comparable to non-respondents concerning age, gender, histology and stage. Almost all variables were distributed equally in respondents and non-respondents (see Table 2).

**Table 1:** Characteristics of patient population

	n	%
Mean age (sd)	66.7	(10.4)
Male	56	60.2
Household size		
Single household	21	22.6
2 Person household	48	51.6
More than 2 person household	24	25.8
Working status		
Retired	57	61.3
Actively working	24	25.8
Unemployed	3	3.2
On sick leave	9	9.7
Education		
Low (≤9 years of school)	36	38.7
Intermediate (10–11 years of school)	30	32.3
High (≥ 12 years of school)	26	28.0
Histology		
NSCLC	81	87.1
Other	12	12.9
Stage at diagnosis		
I	26	28.0
II	12	12.9
III	26	28.0
IV	25	26.9
Information missing	4	4.3
Reason for last visit		
Follow-up care	52	55.9
Oral therapy	10	10.8
Intravenous or radiotherapy	27	29.0
Information missing	4	4.3
Progressive disease	16	17.2
Mean CCI (sd)	4.6	(3.5)
Chronic pulmonary disease	31	33.3
Diabetes	16	17.2
Renal disease	23	24.7
Metastatic disease	42	45.2

Patient characteristics of study population as means with standard deviation for numeric, and relative and absolute frequencies of categorical variables

*Sd* standard deviation, *CCI* Charlson comorbidity index, *NSCLC* non-small cell lung cancer

**Table 2:** Comparison of respondents and non-respondents

	Respondents (n = 93)		Non-respondents (n = 153)		p-value
	Mean	sd	Mean	sd	
Mean age (sd)	66.9	10.4	67.7	12.3	0.71^a^

	n	%	n	%	
Female	40	43.0	67	43.8	1.00^b^
Male	53	57.0	86	56.2	
Histology					
Adenocarcinoma	51	54.8	77	50.3	0.58^b^
Squamous-cell carcinoma	16	17.2	29	19.0	0.86^b^
SCLC	4	4.3	6	3.9	1.00^c^
Mediastinal tumor	3	3.2	9	5.9	0.54^c^
Other	16	17.2	20	13.1	0.48^b^
Unknown	3	3.2	12	7.8	0.23^c^
Stage at diagnosis					
I	26	28.0	43	28.1	1.00^b^
II	12	12.9	17	11.1	0.82^b^
III	26	28.0	32	20.9	0.45^b^
IV	25	26.9	41	26.8	0.66^b^
Unknown	4	4.3	20	13.1	0.02^c^

Comparison of patient characteristics of respondents and non-respondents as means with standard deviation for numeric and relative and absolute frequencies of categorical variables. P-values from Chi^2^ and Fisher-Exact Test (n in cell < 6) for categorical and from T-Test for numerical variables

*Sd* standard deviation, *SCLC* small-cell lung cancer

^a^T-test

^b^Chi^2^-Test

^c^Fisher-Exact Test

### Availability of ambulatory care

14 (15.1%) patients attended ambulatory respiration therapy before the start of the COVID-19 pandemic. For 8 (57.1%) patients this service was unavailable during the height of the pandemic, for one patient it was still unavailable at the time of the study. 20 patients (21.5%) received physical therapy at the beginning of the pandemic, which was unavailable in March and April due to the pandemic for 14 (70%) and available again for 9 patients (45%) at the time of the study. 5 (5.4%) patients participated in a medical sports group and 2 (2.2%) in a self-help group. Both of these services were unavailable in March and April, but available again at the time of completing the questionnaire.

### Inpatient and ambulatory tumor therapy

37 patients (39.8%) had an appointment scheduled for either surgery, chemotherapy, immunotherapy or radiotherapy during March or April. Of these, 7 (18.9%) indicated that this appointment was cancelled (n = 1) or moved to a different date (n = 6) due to the pandemic. The number of days an appointment had been moved was available for three patients and ranged from 3 to 7 days. One patient with stage I had their surgery date moved to a later date, the other 5 patients had stage II or IV disease and indicated their appointments for intravenous therapy or radiotherapy had been moved.

Of the 44 (47.3%) patients with a scheduled appointment for tumor imaging (CT, MRI, PET-CT), 6 (13.6%) had this appointment moved (n = 5) or cancelled (n = 1). The number of days the appointments had been moved was longer compared to tumor therapy and ranged between 5 and 180 days. Four of these patients had stage I or II and were scheduled for follow-up care after curative therapy.

Follow-up care appointments (after surgery or during oral therapy, radiotherapy, chemotherapy or immunotherapy) were planned for 54 (58.1%) patients, and moved (n = 7) or cancelled (n = 1) for 8 (14.8%) of those. If provided, the number of days ranged between 7 and 45 days. This group included mostly patients with follow-up care after curative therapy and patients treated with oral tyrosine kinase inhibitors (TKIs).

### Patient health and behavior

Patients were asked to describe changes to their interpersonal contacts due to COVID-19. More patients indicated limiting contact to friends and acquaintances (48.6%) rather than limiting contact to family members (23.2%). 15.4% of patients said they avoided visits to their primary care physician due to COVID-19, a smaller proportion (5.4%) avoided visits to their pneumologist, oncologist or other specialist. Age, CCI, gender, metastases status were not significantly associated with different levels of agreement. Education level was significantly associated with the statement about meeting family members as well as with visiting their GP, however there was no clear trend in any direction.

The majority of patients did not feel like their general health was affected by the pandemic due to changes in access to medical care (91.4%) or due to the stay-at-home orders (87.1%). Patients who indicated effects on their general health due to restricted access to healthcare or public restrictions were generally younger; however, this difference did not yield significance. Both statements were also not significantly associated with gender, metastatic disease status, education, and CCI.

45.1% of patients indicated that they wore a face mask even in places where it was not mandatory. This was significantly associated with gender (p-value = 0.04), with females being more open to wearing it. However, only 59.8% of patients were confident that they are physically able to wear a face mask over extended periods of time. Around a quarter of all patients felt they experienced shortness of breath or anxiety while wearing a face mask. Age, CCI, metastatic disease status, and education level were not significantly associated with mask wearing behavior.

**Fig. 2:**
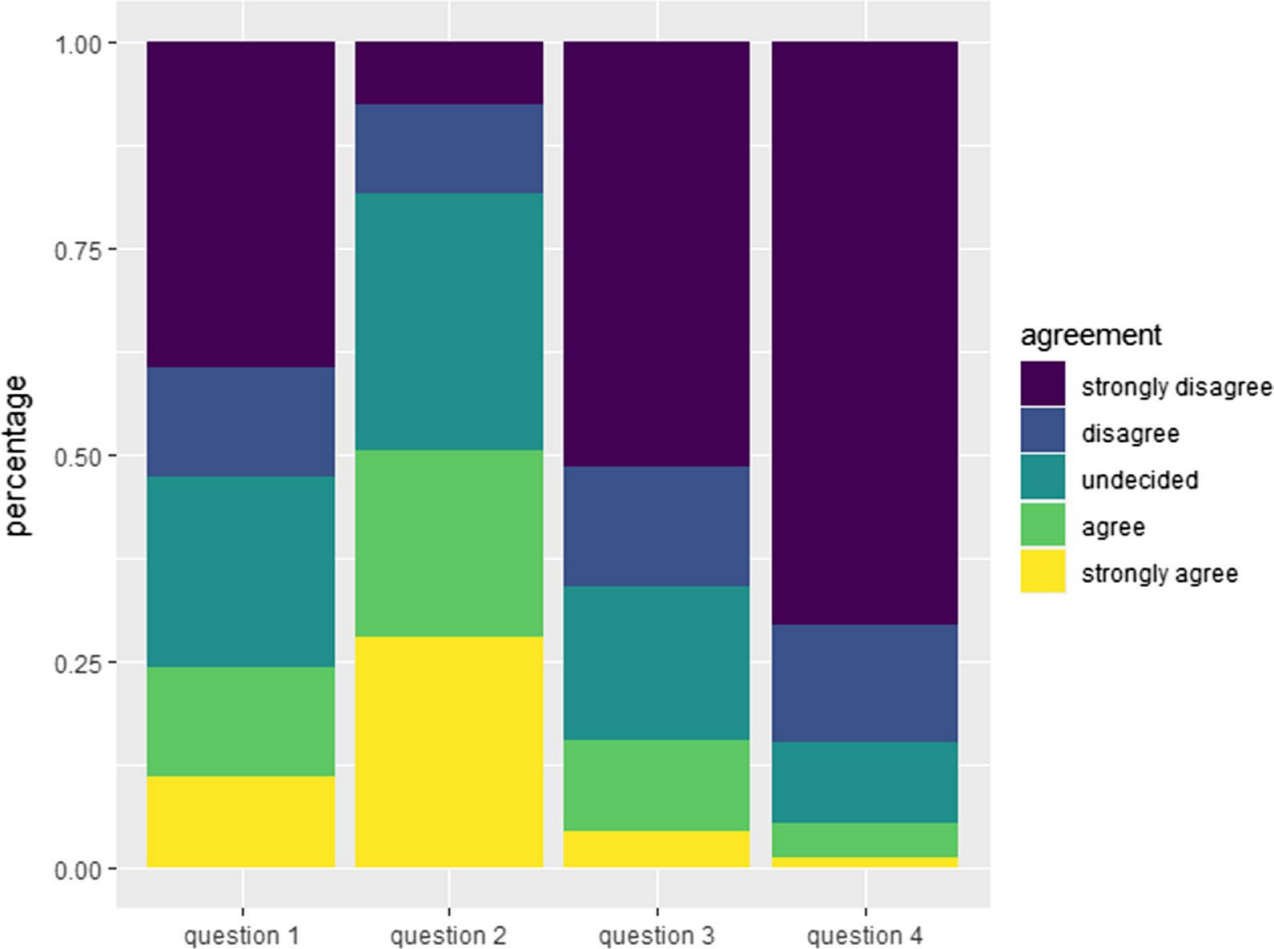
Patient behavior relating to social distancing. Relative frequency of patient answers concerning behavior towards meeting family and friends and visits to their general physician and pneumologist /oncologist or other specialist on a 5-point Likert scale ranging from string agreement to strong disagreement. Question 1: Due to the risk of contracting COVID-19 I avoid meeting family members. Question 2: Due to the risk of contracting COVID-19 I avoid meeting friends and acquaintances. Question 3: Due to the risk of contracting COVID-19 I avoid visits to my primary physician. Question 4: Due to the risk of contracting COVID-19 I avoid visits to my pneumologist/oncologist or other specialists

**Fig. 3:**
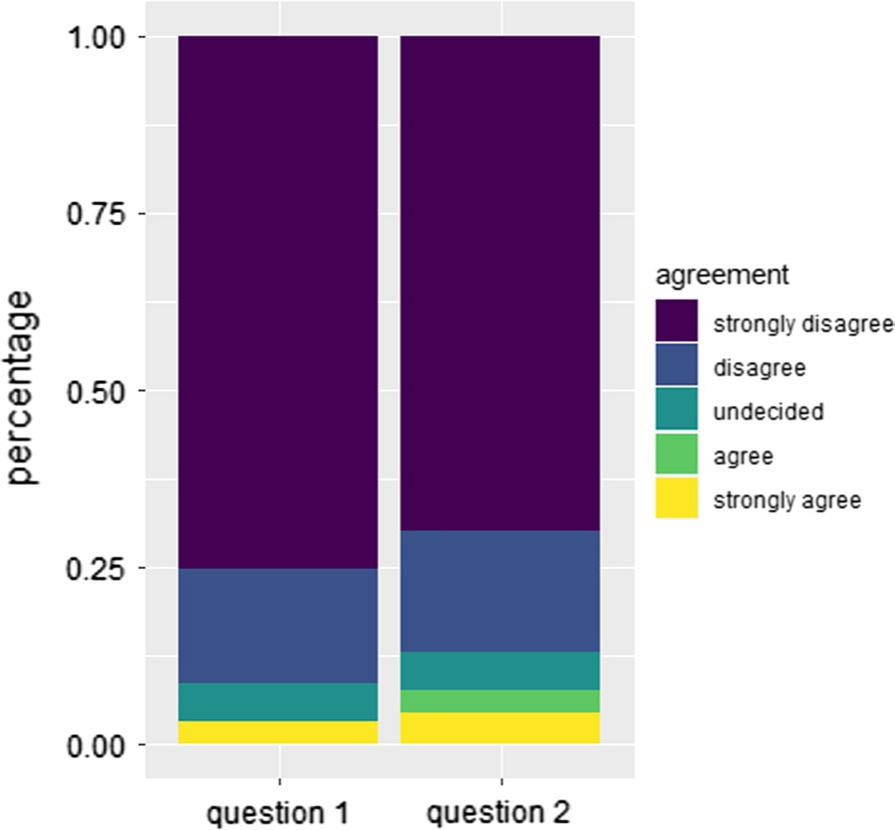
Effects of the stay-at-home order and limited care access on general health. Relative frequency of patient answers concerning possible effects of the limited access to healthcare and the stay-at-home order on patients’ general health on a 5-point Likert scale ranging from string agreement to strong disagreement. Question 1: My general health has declined due to the changes in access to medical care. Question 2: My general health has declined due to the restrictions of the stay-at-home order at the height of the pandemic

**Fig. 4:**
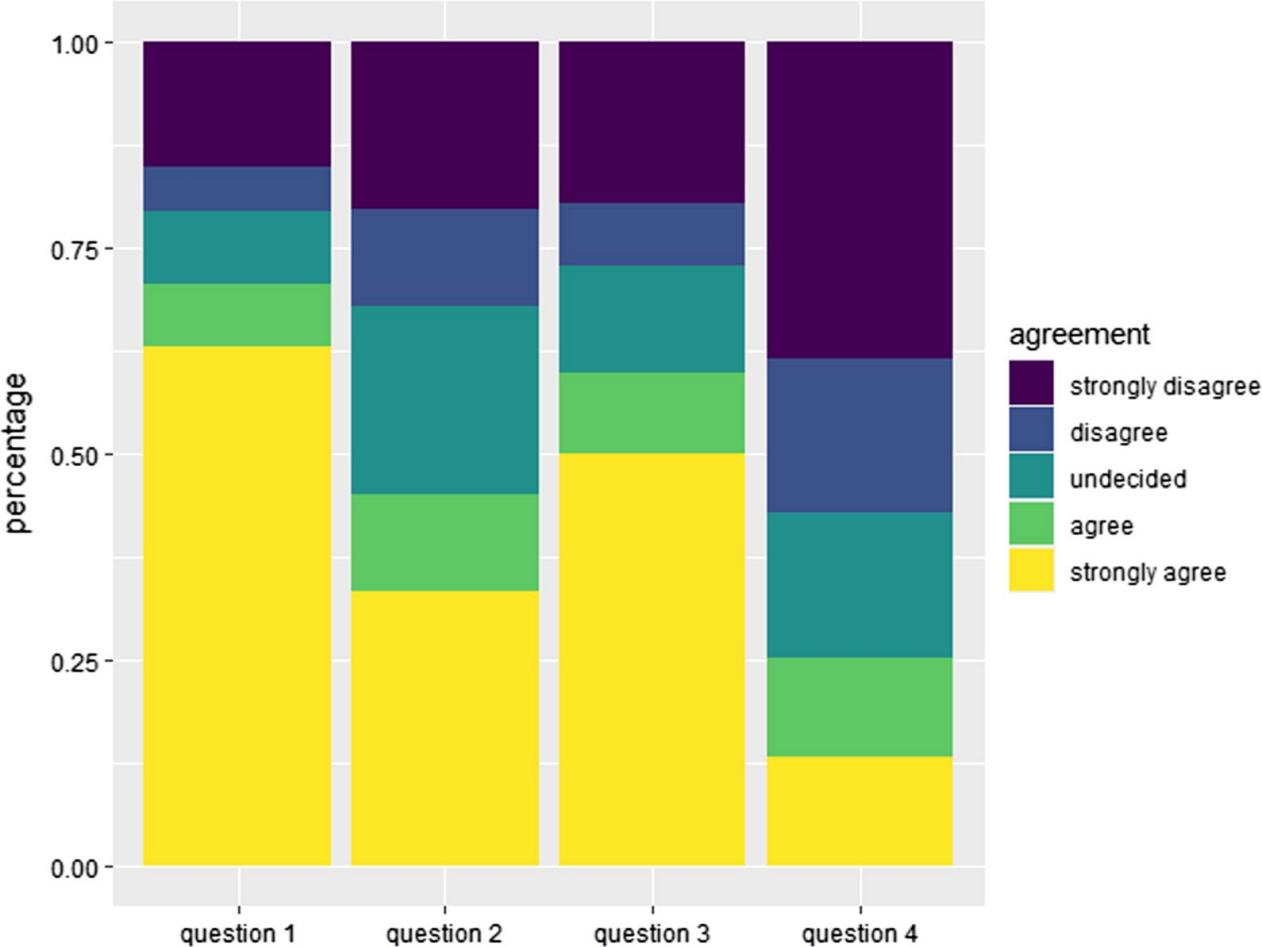
Mask wearing habits and problems when wearing a mask. Relative frequency of patient answers concerning mask wearing habits and problems when wearing a mask on a 5-point Likert scale ranging from string agreement to strong disagreement. Question 1: I only wear my mask in places where it is mandated (e.g. public transportation, supermarket). Question 2: I also wear my mask in places where it is not mandated (e.g. in the park). Question 3: I can wear my mask over a period of 1–2 h without any problems. Question 4: When I wear a facemask I experience shortage of breath/anxiety

Figures 2, 3 and 4 display proportions for all questions with 5 level Likert scale. Additional file 1: Table S1 and Additional file 2: Table S2 display associations with age and CCI, and with gender, metastases status, and education level, respectively.

### COVID-19 exposure, testing, and vaccination

2 patients reported having been in close contact (RKI class I contact) with a person that was infected with COVID-19 at that time. 52 (55.9%) said they had been tested for COVID-19 with a PCR test since the beginning of the pandemic, 9 (9.7%) patients were tested for SARS Cov-2 antibodies. No patients tested positive for COVID-19 or antibodies. The majority of patients (79.6%) said they would agree to get vaccinated against COVID-19. Reasons for not getting vaccinated included fear or insecurity about side effects of the vaccine (4 patients), and fear that the vaccine was developed too quickly (8 patients). 2 patients were still undecided about getting vaccinated. Patients open to getting vaccinated were older (67.7 vs. 63.4) than those unwilling to get vaccinated or those who were not sure yet, however this difference was not significant.

## Discussion

Several surveys regarding experiences of cancer care providers during the pandemic have been published. We believe that this study of patient reported experiences complements and completes the picture presented by the provider perspective. Using paper-based questionnaires, we were able to assess difficulties in access to care as well as exposure to infection and testing of patients with thoracic malignancies (mostly lung cancer) due to the COVID-19 pandemic in Bavaria, Germany. Overall, we found that although some patients (between 13 and 19%) had their appointments cancelled or moved to a different date, the majority of patients received their tumor-directed therapies, tumor imaging, and follow-up care as planned. However, allied health services (e.g. physical therapy, respiratory therapy) were affected to a greater extent and mostly (57–100%) unavailable during the phase of the complete lockdown in March and April. These aspects of care have only slowly started to become available again. Social distancing was not implemented strictly by the majority of patients, with only 49% of patients reporting avoiding meetings with friends and only 23% avoiding seeing family members by the time of the study in October 2020. Only a small proportion of patients (< 10%) said they were avoiding doctor visits, the proportion who did was higher when asked about their GP compared to their pneumologist/oncologist or other specialist. Younger patients more often felt a negative effect of the pandemic on their subjective general health; however, this difference was not significant. Moreover, the majority of patients did not report experiencing negative effects on their general health. The willingness to get vaccinated against SARS-CoV-2 was high (around 80%) and reasons given did not indicate strict refusal but rather insecurity.

A study measuring gaps in cancer treatment due to the COVID-19 pandemic in cancer patients from the Netherlands found that between 12 to 16% of patients experienced postponement of treatments. This finding was similar in our study where 19% of patients reported postponement or cancellation of scheduled appointments for treatments like radiotherapy, chemotherapy, immunotherapy or surgery. Another global study among 54 countries found that 46.3% of oncology centers reported that more than 10% of their patients missed at least one cycle of chemotherapy [7]. When an appointment for tumor therapy was moved, we found that it was moved by a week at the maximum. Therefore, we believe these changes were due to organizational aspects rather than due to the pandemic. The delay in appointments for imaging were greater, reaching 180 days at the maximum. Affected patients were mostly in lower stages, so the risk of recurrence was considered negligible compared to the risk of infection with COVID-19. These findings are in line with ESMO guidelines which allocated high priority for visits for the continuation of in- and outpatient therapies like intravenous chemotherapy and immune therapy as well as radiotherapy, and imaging for patients at a high risk for relapse and to confirm a cancer diagnosis [3]. Lower priority was assigned to follow-up visits for patients with a low or intermediate risk for relapse after radical therapy [3]. The ESMO guidelines also encouraged teleconsultations wherever possible, which seems to be reflected in care, with one survey of 109 representatives from oncology centers reporting that at the peak of the pandemic oncologists used teleconsultations to replace follow-up appointments (94.5%), to monitor oral therapy (92.7%), and to monitor patients receiving immunotherapy (57.8%) or chemotherapy (55%) [13].

Other aspects of allied health care may be more difficult to provide via telemedicine. Exercise such as physical therapy and respiration therapy have been shown to benefit patients with cancer even in advanced stages, increasing control of treatment-related symptoms such as fatigue, and improving patients quality of life [14]. In our study the majority of patients attending such programs reported that these services were not available during the height of the pandemic and were only slowly becoming available again. Newton et al. have published practical recommendations for keeping patients with cancer exercising during the pandemic, including video-communication and in-home exercise [15].

A survey across several European countries measured the willingness to get vaccinated against COVID-19 in more than 7500 people. They found an overall willingness in 74% of respondents, the willingness in Germany was 70% [16]. According to the Germany COVID-19 Snapshot Monitoring (COSMO Germany) the willingness to get vaccinated in the German population has declined from around 79% in April to 50% in October 2020 [17]. In our survey respondents, the willingness to get vaccinated was higher (80%) than the general German population. This higher proportion may reflect thoracic oncology patient perceptions of their individual risk for complications from COVID-19. Additionally, older patients were more likely to indicate a willingness to get vaccinated. Advanced age as well as having a cancer diagnosis are risk factors for complications from COVID-19 [1, 2].

The survey reported here was conducted in a single lung cancer center in Bavaria, Germany. While the results offer a snapshot of the patient experience in Munich, experiences during the pandemic are likely to vary regionally and may also vary between centers in a region. The survey was intended to capture a broad range of patient experiences and was, therefore, mailed out to patients with all types of primary thoracic malignancy and all stages of disease. It included patients with NSCLC, SCLC, mesothelioma and thymus tumors, with patients currently undergoing treatment as well as patients under surveillance and follow-up care. However, as not all surveys were returned, the results may include an element of bias based on patients’ willingness to participate in the survey. However, we did not find that respondents and non-respondents were significantly different regarding age, gender, histology, and stage. However, the definition of our study exclusion criteria may have introduced a selection bias to patients included and excluded for the study. As lung cancer has a median survival time of less than a year and we looked at tumor board records from the previous three years, a higher proportion of patients with lower stage at diagnosis were left to include after excluding deceased patients and patients who were likely deceased. As can be seen in Fig. 1, the majority of patients excluded were patients with metastatic disease who did not visit our center during the 6 months prior to the begin of our study. We expected that these patients most probably were deceased, and we did not want to risk addressing questionnaires to grieving family members. Additionally, as our questionnaire was mailed out only in the German language, there might be an underrepresentation of patients with non-German first language. Nevertheless, apart from stage, basic demographic parameters suggest that the survey respondents were similar to the average lung cancer patient. Mean age of the respondents was 66.7 years which is close to the mean age at diagnosis of German lung cancer patients (male: 69.3, female: 68.3) [18].The proportion of male patients in our study (60%) was comparable to the proportion of male patients among newly diagnosed patients in Germany in 2016 which was 65% [18].

This is the first study to measure specific consequences of the COVID-19 pandemic on ambulatory services as well as in- and outpatient visits from the perspective of thoracic oncology patients in Germany, and, to our knowledge, internationally. Additionally, the study evaluated effects on patient health and patient’s behavior, views and feelings towards social distancing, mask wearing and vaccination. While several studies have reported the physician perspective and described changes to the provision of care, the patient perspective is particularly valuable in demonstrating the effects of those changes, as well as compliance with distancing and lock-down recommendations. In many respects, our results are reassuring and show that, while the pandemic has forced some changes in care, the majority of patients were able to receive most aspects of care as planed and have coped well with the changes that occurred. Other results, such as the relatively high proportion of patients who report not having limited social or family contacts, imply a need for further public health education measures aimed at specific patient groups. Alternatives to mask-wearing should be investigated for those patients who experience respiratory difficulties during extended periods of community mask use.

## Conclusion

This survey of patient experiences demonstrates that the subjective impact of the COVID-19 pandemic on tumor-directed care and general patient health for thoracic oncology patients was minimal. Access to some ambulatory allied health services was greatly reduced. Many patients did not reduce social or family contacts. Most patients wore masks, although many patients reported respiratory symptoms during mask-wearing. The willingness to get vaccinated against SARS-CoV-2 was high. This information is of high relevance to both policy makers and healthcare providers.

## Supplementary Information

**Additional file 1: Table S1. **Supplemental material: Comparison of age and CCI according to degree of agreement.

**Additional file 2: Table S2. **Supplemental material: Comparison of gender, metastatic disease status and education level according to degree of agreement.

## Data Availability

The datasets used and analysed during this current study are available from the corresponding author upon reasonable request.

## Abbreviations

SARS-CoV-2: Severe acute respiratory syndrome corona virus 2
COVID-19: Corona virus disease 2019
ESMO: European Society of Medical Oncology
ICD: International classification of disease
CCI: Charlson comorbidity index
GP: General physician
PCR: Polymerase chain reaction
LCSS: Lung cancer symptom scale
NSCLC: Non-small-cell-lung-cancer
COSMO: COVID-19 snapshot monitoring
SCLC: Small-cell-lung-cancer
RKI: Robert Koch Institut
