# Supplementary material for: Consequences of the COVID-19 pandemic on lung cancer care and patient health in a German lung cancer center: results from a cross-sectional questionnaire
Source: Respir Res. 2022 Jan 29;23:18. doi: 10.1186/s12931-022-01931-z (PMC8799980; doi:10.1186/s12931-022-01931-z)
Supplement: Supplementary file 1 — Additional file 1: Table S1. Supplemental material: Comparison of age and CCI according to degree of agreement. [file 12931_2022_1931_MOESM1_ESM.docx]

**Table 1 Supplemental material: Comparison of age and CCI according to degree of agreement**

| age | strongly agree | | agree | | undecided | | disagree | | strongly disagree | | p-value |
| --- | --- | --- | --- | --- | --- | --- | --- | --- | --- | --- | --- |
|  | mean | sd | mean | sd | mean | sd | mean | sd | mean | sd |  |
| due to the risk of contracting COVID-19 I avoid meeting family members | 61.4 | 12.6 | 64.1 | 7.7 | 66.5 | 12.0 | 69.3 | 7.8 | 68.2 | 10.3 | 0.31 |
| due to the risk of contracting COVID-19 I avoid meeting friends and aquaintances | 66.7 | 10.4 | 62.8 | 9.4 | 69.1 | 10.8 | 68.6 | 10.5 | 66.4 | 10.0 | 0.30 |
| due to the risk of contracting COVID-19 I avoid visits to my primary physician | 71.0 | 6.2 | 61.9 | 8.4 | 63.7 | 11.7 | 68.5 | 11.7 | 68.4 | 9.8 | 0.21 |
| due to the risk of contracting COVID-19 I avoid visits to my pneumologist/oncologist or other specialists | 63.0 | NA | 71.3 | 12.2 | 61.9 | 10.3 | 67.3 | 11.7 | 67.3 | 10.0 | 0.55 |
| my general health has declined due to the changes in access to medical care | 61.0 | 8.2 | NA |  | 66.0 | 9.5 | 71.9 | 7.6 | 65.9 | 10.8 | 0.16 |
| my general health has declined due to the restrictions of the stay-at-home order at the height of the pandemic | 62.8 | 12.4 | 68.0 | 10.8 | 66.6 | 8.7 | 65.6 | 10.6 | 67.2 | 10.5 | 0.92 |
| I only wear my mask in places where it is mandated (e.g. public transportation, supermarket) | 68.5 | 9.9 | 62.7 | 12.0 | 65.0 | 10.1 | 64.8 | 12.8 | 63.8 | 11.0 | 0.45 |
| I also wear my mask in placec where it is not mandated (e.g. in the park, Fussgängerzone) | 67.3 | 11.1 | 64.5 | 9.5 | 67.6 | 11.4 | 64.4 | 10.9 | 67.6 | 8.6 | 0.84 |
| I can wear my mask over a period of 1-2 hours without any problems | 67.7 | 10.0 | 62.0 | 9.1 | 68.3 | 9.5 | 68.9 | 13.9 | 65.5 | 10.8 | 0.54 |
| when I wear a face-mask I experience shortage of breath/anxiety | 67.5 | 12.9 | 68.7 | 9.5 | 66.8 | 9.9 | 68.3 | 11.0 | 64.8 | 10.0 | 0.73 |
| CCI | strongly agree | | agree | | undecided | | disagree | | strongly disagree | | p-value |
|  | mean | sd | mean | sd | mean | sd | mean | sd | mean | sd |  |
| due to the risk of contracting COVID-19 I avoid meeting family members | 3.6 | 3.1 | 4.9 | 3.4 | 4.1 | 3.3 | 3.9 | 3.9 | 5.4 | 3.6 | 0.45 |
| due to the risk of contracting COVID-19 I avoid meeting friends and aquaintances | 4.9 | 3.0 | 4.9 | 3.8 | 4.5 | 3.7 | 4.2 | 3.7 | 3.9 | 3.5 | 0.94 |
| due to the risk of contracting COVID-19 I avoid visits to my primary physician | 4.5 | 4.0 | 4.8 | 3.5 | 6.1 | 3.0 | 3.9 | 2.8 | 4.3 | 3.7 | 0.41 |
| due to the risk of contracting COVID-19 I avoid visits to my pneumologist/oncologist or other specialists | 5.0 | NA | 4.0 | 2.9 | 5.3 | 2.5 | 2.8 | 2.5 | 5.0 | 3.7 | 0.31 |
| my general health has declined due to the changes in access to medical care | 4.7 | 2.5 | NA | NA | 4.8 | 5.3 | 4.7 | 3.4 | 4.6 | 3.5 | 1.00 |
| my general health has declined due to the restrictions of the stay-at-home order at the height of the pandemic | 6.3 | 3.8 | 6.3 | 4.5 | 5.0 | 3.8 | 4.3 | 3.2 | 4.5 | 3.5 | 0.75 |
| I only wear my mask in places where it is mandated (e.g. public transportation, supermarket) | 4.8 | 3.6 | 3.6 | 3.3 | 4.1 | 3.1 | 4.8 | 3.6 | 4.9 | 3.4 | 0.75 |
| I also wear my mask in placec where it is not mandated (e.g. in the park, Fussgängerzone) | 5.1 | 3.3 | 5.6 | 3.6 | 4.4 | 3.5 | 4.1 | 3.2 | 3.6 | 3.8 | 0.51 |
| I can wear my mask over a period of 1-2 hours without any problems | 4.8 | 3.5 | 4.3 | 3.9 | 5.4 | 3.9 | 3.6 | 3.9 | 4.3 | 2.9 | 0.81 |
| when I wear a face-mask I experience shortage of breath/anxiety | 4.8 | 3.4 | 4.2 | 2.9 | 4.6 | 3.9 | 4.8 | 3.3 | 4.7 | 3.8 | 0.99 |

Notes: Mean values and standard deviation of age and CCI, across different levels of agreements with statements concerning social distancing and mask wearing. P-values from ANOVA.

CCI = Charlson comorbidity index, sd = standard deviation, ANOVA = analysis of variance.
