# Supplementary material for: Consequences of the COVID-19 pandemic on lung cancer care and patient health in a German lung cancer center: results from a cross-sectional questionnaire
Source: Respir Res. 2022 Jan 29;23:18. doi: 10.1186/s12931-022-01931-z (PMC8799980; doi:10.1186/s12931-022-01931-z)
Supplement: Supplementary file 2 — Additional file 2: Table S2. Supplemental material: Comparison of gender, metastatic disease status and education level according to degree of agreement. [file 12931_2022_1931_MOESM2_ESM.docx]

**Table 2 Supplemental material: Comparison of gender, metastatic disease status and education level according to degree of agreement**

| male |  | strongly agree | | agree | | undecided | | disagree | | strongly disagree | | p-value |
| --- | --- | --- | --- | --- | --- | --- | --- | --- | --- | --- | --- | --- |
|  |  | n | % | n | % | n | % | n | % | n | % |  |
| due to the risk of contracting COVID-19 I avoid meeting family members |  | 4 | 40.0% | 8 | 66.7% | 13 | 61.9% | 10 | 83.3% | 20 | 55.6% | 0.29 |
| due to the risk of contracting COVID-19 I avoid meeting friends and aquaintances |  | 17 | 65.4% | 10 | 47.6% | 20 | 69.0% | 5 | 50.0% | 4 | 57.1% | 0.53 |
| due to the risk of contracting COVID-19 I avoid visits to my primary physician |  | 3 | 75.0% | 6 | 60.0% | 7 | 41.2% | 8 | 61.5% | 31 | 66.0% | 0.47 |
| due to the risk of contracting COVID-19 I avoid visits to my pneumologist/oncologist or other specialists |  | 1 | 100.0% | 3 | 75.0% | 3 | 33.3% | 7 | 53.8% | 42 | 64.6% | 0.34 |
| my general health has declined due to the changes in access to medical care |  | 1 | 33.3% | 0 | 0.0% | 2 | 40.0% | 11 | 73.3% | 42 | 60.0% | 0.41 |
| my general health has declined due to the restrictions of the stay-at-home order at the height of the pandemic |  | 2 | 50.0% | 0 | 0.0% | 2 | 40.0% | 11 | 68.8% | 41 | 63.1% | 0.20 |
| I only wear my mask in places where it is mandated (e.g. public transportation, supermarket) |  | 37 | 63.8% | 2 | 28.6% | 5 | 62.5% | 3 | 60.0% | 9 | 64.3% | 0.45 |
| I also wear my mask in placec where it is not mandated (e.g. in the park, Fussgängerzone) |  | 13 | 41.9% | 5 | 45.5% | 15 | 71.4% | 8 | 72.7% | 15 | 78.9% | **0.04** |
| I can wear my mask over a period of 1-2 hours without any problems |  | 29 | 63.0% | 5 | 55.6% | 6 | 50.0% | 5 | 71.4% | 11 | 61.1% | 0.91 |
| when I wear a face-mask I experience shortage of breath/anxiety |  | 8 | 66.7% | 7 | 63.6% | 9 | 56.3% | 10 | 58.8% | 21 | 60.0% | 0.99 |
| metastatic disease |  | strongly agree | | agree | | undecided | | disagree | | strongly disagree | | p-value |
|  |  | mean | sd | mean | sd | mean | sd | mean | sd | mean | sd |  |
| due to the risk of contracting COVID-19 I avoid meeting family members |  | 2 | 20.0% | 7 | 58.3% | 7 | 33.3% | 1 | 8.3% | 9 | 27.3% | 0.11 |
| due to the risk of contracting COVID-19 I avoid meeting friends and aquaintances |  | 9 | 34.6% | 4 | 19.0% | 11 | 39.3% | 1 | 10.0% | 1 | 20.0% | 0.33 |
| due to the risk of contracting COVID-19 I avoid visits to my primary physician |  | 1 | 25.0% | 3 | 30.0% | 4 | 26.7% | 5 | 38.5% | 13 | 28.3% | 0.96 |
| due to the risk of contracting COVID-19 I avoid visits to my pneumologist/oncologist or other specialists |  | 0 | 0.0% | 1 | 25.0% | 1 | 12.5% | 3 | 25.0% | 21 | 32.8% | 0.83 |
| my general health has declined due to the changes in access to medical care |  | 0 | 0.0% | 0 | 0.0% | 0 | 0.0% | 3 | 21.4% | 23 | 33.3% | 0.40 |
| my general health has declined due to the restrictions of the stay-at-home order at the height of the pandemic |  | 0 | 0.0% | 0 | 0.0% | 0 | 0.0% | 5 | 33.3% | 21 | 32.8% | 0.41 |
| I only wear my mask in places where it is mandated (e.g. public transportation, supermarket) |  | 14 | 25.0% | 0 | 0.0% | 4 | 50.0% | 1 | 20.0% | 7 | 50.0% | 0.12 |
| I also wear my mask in placec where it is not mandated (e.g. in the park, Fussgängerzone) |  | 13 | 41.9% | 4 | 40.0% | 3 | 14.3% | 3 | 27.3% | 3 | 17.6% | 0.15 |
| I can wear my mask over a period of 1-2 hours without any problems |  | 16 | 34.8% | 2 | 22.2% | 1 | 8.3% | 2 | 33.3% | 5 | 31.3% | 0.47 |
| when I wear a face-mask I experience shortage of breath/anxiety |  | 2 | 20.0% | 5 | 50.0% | 2 | 12.5% | 3 | 17.6% | 13 | 37.1% | 0.17 |
| education |  | strongly agree | | agree | | undecided | | disagree | | strongly disagree | | p-value |
|  |  | n | % | n | % | n | % | n | % | n | % |  |
| due to the risk of contracting COVID-19 I avoid meeting family members | low | 5 | 50.0% | 1 | 8.3% | 9 | 45.0% | 8 | 66.7% | 12 | 33.3% | **0.03** |
|  | medium | 4 | 40.0% | 3 | 25.0% | 8 | 40.0% | 1 | 8.3% | 13 | 36.1% |  |
|  | high | 1 | 10.0% | 8 | 66.7% | 3 | 15.0% | 3 | 25.0% | 11 | 30.6% |  |
| due to the risk of contracting COVID-19 I avoid meeting friends and aquaintances | low | 8 | 32.0% | 6 | 28.6% | 15 | 51.7% | 4 | 57.1% | 3 | 42.9% | 0.13 |
|  | medium | 9 | 36.0% | 10 | 47.6% | 8 | 27.6% | 0 | 0.0% | 3 | 42.9% |  |
|  | high | 8 | 32.0% | 5 | 23.8% | 6 | 20.7% | 3 | 42.9% | 1 | 14.3% |  |
| due to the risk of contracting COVID-19 I avoid visits to my primary physician | low | 2 | 50.0% | 1 | 10.0% | 6 | 37.5% | 4 | 30.8% | 23 | 48.9% | **0.004** |
|  | medium | 0 | 0.0% | 6 | 60.0% | 10 | 62.5% | 3 | 23.1% | 10 | 21.3% |  |
|  | high | 2 | 50.0% | 3 | 30.0% | 0 | 0.0% | 6 | 46.2% | 14 | 29.8% |  |
| due to the risk of contracting COVID-19 I avoid visits to my pneumologist/oncologist or other specialists | low | 1 | 100.0% | 2 | 50.0% | 2 | 25.0% | 4 | 30.8% | 27 | 41.5% | 0.53 |
|  | medium | 0 | 0.0% | 0 | 0.0% | 5 | 62.5% | 5 | 38.5% | 20 | 30.8% |  |
|  | high | 0 | 0.0% | 2 | 50.0% | 1 | 12.5% | 4 | 30.8% | 18 | 27.7% |  |
| my general health has declined due to the changes in access to medical care | low | 3 | 100.0% | 0 | 0.0% | 2 | 40.0% | 6 | 40.0% | 25 | 36.2% | 0.22 |
|  | medium | 0 | 0.0% | 0 | 0.0% | 3 | 60.0% | 3 | 20.0% | 24 | 34.8% |  |
|  | high | 0 | 0.0% | 0 | 0.0% | 0 | 0.0% | 6 | 40.0% | 20 | 29.0% |  |
| my general health has declined due to the restrictions of the stay-at-home order at the height of the pandemic | low | 3 | 75.0% | 1 | 33.3% | 0 | 0.0% | 9 | 56.3% | 23 | 35.9% | 0.31 |
|  | medium | 1 | 25.0% | 1 | 33.3% | 2 | 40.0% | 4 | 25.0% | 22 | 34.4% |  |
|  | high | 0 | 0.0% | 1 | 33.3% | 3 | 60.0% | 3 | 18.8% | 19 | 29.7% |  |
| I only wear my mask in places where it is mandated (e.g. public transportation, supermarket) | low | 25 | 43.9% | 3 | 42.9% | 2 | 25.0% | 0 | 0.0% | 6 | 42.9% | 0.46 |
|  | medium | 18 | 31.6% | 3 | 42.9% | 2 | 25.0% | 2 | 40.0% | 5 | 35.7% |  |
|  | high | 14 | 24.6% | 1 | 14.3% | 4 | 50.0% | 3 | 60.0% | 3 | 21.4% |  |
| I also wear my mask in placec where it is not mandated (e.g. in the park, Fussgängerzone) | low | 13 | 41.9% | 3 | 27.3% | 6 | 30.0% | 6 | 55.8% | 8 | 42.1% | 0.82 |
|  | medium | 9 | 29.0% | 6 | 54.5% | 7 | 35.0% | 3 | 26.5% | 5 | 26.3% |  |
|  | high | 9 | 29.0% | 2 | 18.2% | 7 | 35.0% | 2 | 17.7% | 6 | 31.6% |  |
| I can wear my mask over a period of 1-2 hours without any problems | low | 16 | 35.6% | 2 | 22.2% | 7 | 58.3% | 4 | 57.1% | 7 | 38.9% | 0.37 |
|  | medium | 16 | 35.6% | 6 | 66.7% | 3 | 25.0% | 1 | 14.3% | 4 | 22.2% |  |
|  | high | 13 | 28.9% | 1 | 11.1% | 2 | 16.7% | 2 | 28.6% | 7 | 38.9% |  |
| when I wear a face-mask I experience shortage of breath/anxiety | low | 6 | 50.0% | 2 | 18.2% | 7 | 43.8% | 9 | 52.9% | 11 | 32.4% | 0.52 |
|  | medium | 3 | 25.0% | 3 | 27.3% | 6 | 37.5% | 4 | 23.5% | 14 | 41.2% |  |
|  | high | 3 | 25.0% | 6 | 54.5% | 3 | 18.8% | 4 | 23.5% | 9 | 26.5% |  |

Notes: Absolute and relative frequencies of gender, metastatic disease status, and education level, across different levels of agreements with statements concerning social distancing and mask wearing. P-values from Chi^2^-Test and Fisher Exact Test.
